# Supplementary material for: Decomposition of phenotypic heterogeneity in autism reveals underlying genetic programs
Source: Nat Genet. 2025 Jul 9;57(7):1611–9. doi: 10.1038/s41588-025-02224-z (PMC12283356; doi:10.1038/s41588-025-02224-z)
Supplement: Supplementary file 1 — Supplementary Note. [file 41588_2025_2224_MOESM1_ESM.pdf]

nature genetics

Supplementary information https://doi.org/10.1038/s41588-025-02224-z

Decomposition of phenotypic heterogeneity in autism reveals underlying genetic programs

In the format provided by the authors and unedited

Table of Contents

1. **Supplementary Note: Pages 2-3**

Supplementary Note

Phenotype measures included in the model

We applied a finite mixture modeling analysis based on the phenotype features described above. The Background history form provided information on numerous developmental milestones including, but not limited to, age first walked, age first talked, age when combined phrases, age when bladder trained, etc. These features are numerical and measure parent-reported age (in months) when developmental milestones were achieved. The SCQ is a widely-used measure covering primarily social behavior difficulties and social communication, and repetitive/restrictive behaviors to a lesser extent. The questionnaire consists of 40 binary questions pertaining to social behaviors, communication, social interaction, sensory experiences, etc. The total score ranges from 0-39, with a higher score indicating higher impairment (more difficulties). We excluded the first question of the SCQ (q01_phrases), which is not used in the calculation of the total score. The RBS-R is a questionnaire covering six factors of behavior: self-injury, sameness, restrictive, repetitive, stereotyped, and ritualistic behaviors. It includes 43 categorical questions (rated 0-3) which load into six composite scores covering each area of behavior. The total score for the RBS-R ranges from 0-129, where higher scores indicate higher impairment (more difficulties). Finally, the CBCL 6-18 is a rating scale with 144 questions (121 of which were implemented in this study due to the exclusion of text-based questions) covering many co-occurring traits and conditions, including ADHD, OCD, depression, anxiety, somatic problems, etc. This questionnaire only covers individuals between 6 and 18 years of age; however, some individuals whose age is marked as less than 6 years old in our cohort had this measure available due to later administration of the test after recruitment. CBCL questions are measured on a scale from 0-2, where higher scores indicate more frequent behavior. These are summed up into 20 standardized composite t-scores each ranging from 0-100.

Exploratory class enumeration indicators

The LL, AIC, CAIC, BIC, and CABIC (Supplementary Fig. 1) were computed over 200 independent runs and can be evaluated according to the elbow criterion: we looked for the point where the marginal gains in fit are diminished, causing an elbow in the plot. This point is located around 4-6 components in all five plots. The LL was computed using a cross-validation scheme with 3 folds, where a model was fit on ⅔ of the cohort data, and tested on the remaining ⅓ of the data. The log likelihood was then computed and summed across individuals in each class. The Lo-Mendell-Rubin Likelihood Ratio Test (LMR-LRT) was performed over 50 independent runs of the model with randomly generated seeds. The LMR-LRT results were evaluated according to the point where the average p-value increases above the cutoff for

significance (alpha), marking the model with the best balance of fit and complexity. For our data, this point clearly occurred at n_components = 4, suggesting worse fit for the 5 and 6 component models as compared to the 4 component model. We additionally measured average posterior probability (AvePP), entropy, and count and proportion of the smallest class. Neither AvePP nor entropy were used as measures for model selection, but rather as confirmation of model fit. A model with a higher number of components tends towards fragmentation of class sizes, so we sought to balance meaningful class sizes with adequate phenotypic decomposition.

Naming

We consulted with clinicians, parents of autistic children, autistic adults, and other researchers to determine naming that would be accurate, descriptive, and non-ableist.
