# Supplementary material for: Decomposition of phenotypic heterogeneity in autism reveals underlying genetic programs
Source: Nat Genet. 2025 Jul 9;57(7):1611–9. doi: 10.1038/s41588-025-02224-z (PMC12283356; doi:10.1038/s41588-025-02224-z)
Supplement: Supplementary file 2 — Reporting Summary [file 41588_2025_2224_MOESM2_ESM.pdf]

nature portfolio

Corresponding author(s): Olga Troyanskaya
Last updated by author(s): 04/28/2025

# Reporting Summary

Nature Portfolio wishes to improve the reproducibility of the work that we publish. This form provides structure for consistency and transparency in reporting. For further information on Nature Portfolio policies, see our Editorial Policies and the Editorial Policy Checklist.

## Statistics

For all statistical analyses, confirm that the following items are present in the figure legend, table legend, main text, or Methods section.

| n/a | Confirmed | |
|---|---|---|
| ☐ | ☒ | The exact sample size (*n*) for each experimental group/condition, given as a discrete number and unit of measurement |
| ☐ | ☒ | A statement on whether measurements were taken from distinct samples or whether the same sample was measured repeatedly |
| ☐ | ☒ | The statistical test(s) used AND whether they are one- or two-sided<br>*Only common tests should be described solely by name; describe more complex techniques in the Methods section.* |
| ☐ | ☒ | A description of all covariates tested |
| ☐ | ☒ | A description of any assumptions or corrections, such as tests of normality and adjustment for multiple comparisons |
| ☐ | ☒ | A full description of the statistical parameters including central tendency (e.g. means) or other basic estimates (e.g. regression coefficient) AND variation (e.g. standard deviation) or associated estimates of uncertainty (e.g. confidence intervals) |
| ☐ | ☒ | For null hypothesis testing, the test statistic (e.g. *F*, *t*, *r*) with confidence intervals, effect sizes, degrees of freedom and *P* value noted<br>*Give P values as exact values whenever suitable.* |
| ☐ | ☒ | For Bayesian analysis, information on the choice of priors and Markov chain Monte Carlo settings |
| ☐ | ☒ | For hierarchical and complex designs, identification of the appropriate level for tests and full reporting of outcomes |
| ☐ | ☒ | Estimates of effect sizes (e.g. Cohen's *d*, Pearson's *r*), indicating how they were calculated |

*Our web collection on statistics for biologists contains articles on many of the points above.*

## Software and code

Policy information about availability of computer code

| | |
|---|---|
| Data collection | No software was used to collect data. |
| Data analysis | HAT (no version number but downloaded from GitHub on 10/11/22) was used for variant calling.<br>plink v1.9 was used to compute polygenic scores.<br>ShinyGO 0.80 was used to perform GO term enrichment analyses.<br>StepMix 1.2.5 was retrieved from PyPI and used to construct and train the mixture models.<br>Ensembl VEP (Release 111.0) was used to call variant effects for rare coding variants.<br>LOFTEE (v.1.0.4) was used to call loss-of-function variants.<br>AlphaMissense (VEP plugin) was used to call missense variants.<br>DeepVariant (v1.1.0) was used for variant calling.<br>GATK HaplotypeCaller (v4.1.2.0) was used for variant calling. |

For manuscripts utilizing custom algorithms or software that are central to the research but not yet described in published literature, software must be made available to editors and reviewers. We strongly encourage code deposition in a community repository (e.g. GitHub). See the Nature Portfolio guidelines for submitting code & software for further information.

## Data

Policy information about availability of data

All manuscripts must include a data availability statement. This statement should provide the following information, where applicable:

- Accession codes, unique identifiers, or web links for publicly available datasets
- A description of any restrictions on data availability
- For clinical datasets or third party data, please ensure that the statement adheres to our policy

In order to abide by the informed consents that individuals with autism and their family members signed when agreeing to participate in a SFARI cohort (SSC and SPARK), researchers must be approved by SFARI Base (https://base.sfari.org).

## Research involving human participants, their data, or biological material

Policy information about studies with human participants or human data. See also policy information about sex, gender (identity/presentation), and sexual orientation and race, ethnicity and racism.

| | |
|---|---|
| Reporting on sex and gender | SPARK Consortium. Electronic address: pfeliciano@simonsfoundation.org & SPARK Consortium. SPARK: A US Cohort of 50,000 Families to Accelerate Autism Research. Neuron 97, 488–493 (2018). |
| Reporting on race, ethnicity, or other socially relevant groupings | SPARK Consortium. Electronic address: pfeliciano@simonsfoundation.org & SPARK Consortium. SPARK: A US Cohort of 50,000 Families to Accelerate Autism Research. Neuron 97, 488–493 (2018). |
| Population characteristics | The study cohort consisted of 5,392 children with a professional autism diagnosis aged 4-18 years (mean = 8.56 years, SD = 3.15). The control population consisted of 1,972 siblings without a professional diagnosis of autism aged 4-18 years (mean = 7.95 years, SD = 4.41). In these populations, 4,636 probands and 1,972 siblings had whole exome sequencing (WES) data available.<br><br>Additional details can be found in: SPARK Consortium. Electronic address: pfeliciano@simonsfoundation.org & SPARK Consortium. SPARK: A US Cohort of 50,000 Families to Accelerate Autism Research. Neuron 97, 488–493 (2018). |
| Recruitment | SPARK Consortium. Electronic address: pfeliciano@simonsfoundation.org & SPARK Consortium. SPARK: A US Cohort of 50,000 Families to Accelerate Autism Research. Neuron 97, 488–493 (2018). |
| Ethics oversight | We received approval to access and analyze de-identified genetic and phenotypic data from the two cohorts from SFARI Base and the Princeton University IRB Committee in the Office of Research Integrity. |

Note that full information on the approval of the study protocol must also be provided in the manuscript.

# Field-specific reporting

Please select the one below that is the best fit for your research. If you are not sure, read the appropriate sections before making your selection.

☒ Life sciences ☐ Behavioural & social sciences ☐ Ecological, evolutionary & environmental sciences

For a reference copy of the document with all sections, see nature.com/documents/nr-reporting-summary-flat.pdf

# Life sciences study design

All studies must disclose on these points even when the disclosure is negative.

| | |
|---|---|
| Sample size | n = 5,392 (SPARK proband cohort), n = 1,972 (SPARK sibling cohort), and n = 861 (SSC proband cohort). Measures in SPARK with sufficiently high overlap among participants were selected to maximize the cohort size while maintaining breadth of phenotype data. All probands with complete or mostly complete data for four phenotype battery assays (Background History, Social Communication Questionnaire, Repetitive Behavior Scale, and Child Behavior Checklist) were included in the analysis. More details are available in the Methods section. Extensive statistical testing for both phenotypic and genetic analyses, including adjustment for multiple hypotheses, validated the sufficiency of these sample sizes. |
| Data exclusions | Data exclusion was performed based on availability and completion of data. Every participant who had the required data available (mostly complete across four phenotype battery assays) was included. Additionally, for genetic analyses of de novo variation, we excluded individuals who had an outlier count of de novo variants (defined as 3 standard deviations above the mean de novo count across the sample). |
| Replication | Replication was performed on a phenotype dataset from another cohort (the Simons Simplex Collection) which included n = 861 participants with complete phenotype information for four phenotype assays. Multiple replication methods were tested and all replication attempts were successful. More details on replication are available in the Methods section. |
| Randomization | Covariates were controlled for in the main mixture model used to classify the sample. Covariates include sex and age at evaluation as |

| | |
|---|---|
| Randomization | provided by the phenotype dataset. |
| Blinding | This study is a computational analysis of data-drive subclasses of autism. We received approval to analyze SPARK and SSC data from SFARI Base, and we had no contact with study participants. No treatment was administered, and therefore blinding is not applicable to this line of data-driven analysis. |

# Reporting for specific materials, systems and methods

We require information from authors about some types of materials, experimental systems and methods used in many studies. Here, indicate whether each material, system or method listed is relevant to your study. If you are not sure if a list item applies to your research, read the appropriate section before selecting a response.

## Materials & experimental systems

| n/a | Involved in the study | |
|---|---|---|
| ☒ | ☐ | Antibodies |
| ☒ | ☐ | Eukaryotic cell lines |
| ☒ | ☐ | Palaeontology and archaeology |
| ☒ | ☐ | Animals and other organisms |
| ☒ | ☐ | Clinical data |
| ☒ | ☐ | Dual use research of concern |
| ☒ | ☐ | Plants |

## Methods

| n/a | Involved in the study | |
|---|---|---|
| ☒ | ☐ | ChIP-seq |
| ☒ | ☐ | Flow cytometry |
| ☒ | ☐ | MRI-based neuroimaging |

## Plants

| | |
|---|---|
| Seed stocks | *Report on the source of all seed stocks or other plant material used. If applicable, state the seed stock centre and catalogue number. If plant specimens were collected from the field, describe the collection location, date and sampling procedures.* |
| Novel plant genotypes | *Describe the methods by which all novel plant genotypes were produced. This includes those generated by transgenic approaches, gene editing, chemical/radiation-based mutagenesis and hybridization. For transgenic lines, describe the transformation method, the number of independent lines analyzed and the generation upon which experiments were performed. For gene-edited lines, describe the editor used, the endogenous sequence targeted for editing, the targeting guide RNA sequence (if applicable) and how the editor was applied.* |
| Authentication | *Describe any authentication procedures for each seed stock used or novel genotype generated. Describe any experiments used to assess the effect of a mutation and, where applicable, how potential secondary effects (e.g. second site T-DNA insertions, mosiacism, off-target gene editing) were examined.* |
